# The Generative Mechanisms of Financial Strain and Financial Well-Being: A Critical Realist Analysis of Ideology and Difference

**DOI:** 10.34172/ijhpm.2022.6930

**Published:** 2022-12-06

**Authors:** Nicole M. Glenn, Aryati Yashadhana, Karla Jaques, Ana Belon, Evelyne de Leeuw, Candace I. J. Nykiforuk, Patrick Harris

**Affiliations:** ^1^Centre for Healthy Communities, School of Public Health, University of Alberta, Edmonton, AB, Canada.; ^2^PolicyWise for Children & Families, Edmonton, AB, Canada.; ^3^Centre for Health Equity Training Research & Evaluation (CHETRE), University of New South Wales, Sydney, NSW, Australia.; ^4^Centre for Primary Health Care and Equity, University of New South Wales, Sydney, NSW, Australia.; ^5^Ingham Institute for Applied Medical Research, Sydney, NSW, Australia.; ^6^School of Social Sciences, University of New South Wales, Sydney, NSW, Australia.

**Keywords:** Critical Realism, Financial Well-Being, Financial Strain, COVID-19, Inequities, Public Health

## Abstract

**Background:** Rapid, strategic action is required to mitigate the negative and unequal impact of the coronavirus disease 2019 (COVID-19) pandemic on the financial well-being (FWB) of global populations. Personal financial strain (FS) worsened most significantly among systematically excluded groups. Targeted government- and community-led initiatives are needed to address these inequities. The purpose of this applied research was to identify what works for whom, under what conditions, and why in relation to community and government initiatives that promote personal and household FWB and/or address FS in high income economies.

**Methods:** We employed a critical realist analysis to literature that reported on FWB/FS initiatives in high income countries. This included initiatives introduced in response to the pandemic as well as those that began prior to the pandemic. We included sources based on a rapid review. We coded academic, published literature (n=39) and practice-based (n=36) reports abductively to uncover generative mechanisms – ie, underlying, foundational factors related to community or government initiatives that either constrained and/or enabled FWB and FS.

**Results:** We identified two generative mechanisms: (1) neoliberal ideology; and (2) social equity ideology. A third mechanism, social location (eg, characteristics of identity, location of residence), cut across the two ideologies and demonstrated for whom the initiatives worked (or did not) in what circumstances. Neoliberal ideology (ie, individual responsibility) dominated initiative designs, which limited the positive impact on FS. This was particularly true for people who occupied systematically excluded social locations (eg, low-income young mothers). Social equity-based initiatives were less common within the literature, yet mostly had a positive impact on FWB and produced equitable outcomes.

**Conclusion:** Equity-centric initiatives are required to improve FWB and reduce FS among systemically excluded and marginalized groups. These findings are of relevance now as nations strive for financial recovery in the face of the ongoing global pandemic.

## Background

**Key Messages**

** Implications for policy makers**

- This paper reviews what works for whom, under what conditions, and why in relation to initiatives that promote personal and household financial well-being (FWB) and/or address financial strain (FS) in high-income economies. The findings can inform personal/household economic recovery-focused policies and practices in response to coronavirus disease 2019 (COVID-19).
- Policies embedded in neoliberal ideology, such as individualized behaviour change initiatives, had limited positive impact on personal or household long-term FWB and/or FS. Such policies were particularly ineffective at improving equity in health, social, and economic outcomes.
- Policies that took an equity-targeted approach, such as the strengthening of social welfare protection and the reduction of contingencies, demonstrated positive impacts on FWB and FS among systemically excluded populations.

** Implications for the public**

Many people across the globe have felt the negative impacts of coronavirus disease 2019 (COVID-19) on their financial well-being (FWB). In this paper, we review initiatives like government labour, housing, and social welfare policies as well as community or organization programs like money management classes, to understand what works and does not work, for whom, and under which circumstances to improve FWB and financial strain (FS). We include initiatives introduced in response to the pandemic as well as those active prior to its start. Our findings can help policy-makers and program-providers to create initiatives that improve people’s financial circumstances and reduce FS in the aftermath of COVID-19. We highlight how social-equity-focused initiatives can promote personal and household FWB among systemically excluded groups.

The pandemic has led to unprecedented governmental action on financial strain (FS) and financial well-being (FWB) across the globe.^1^ For example, in high-income contexts, governments introduced increases in social welfare-based cash benefits,^2^ rental freezes and restrictions on evictions,^3^ and strengthened employment protection,^1,3,4^ among others.^3^ Many of these initiatives have since ceased or scaled back as countries have entered ‘recovery’ mode.^5^ To ensure an equitable economic recovery from coronavirus disease 2019 (COVID-19), research on what FS and FWB initiatives work for whom under which circumstances is required. To contribute to this body of evidence, we examined the foundational factors of initiatives (eg, policies, programs) that constrain or enable personal FWB or FS (ie, generative mechanisms). We included in our investigation emergent literature focused on the equity and financial impact of initiatives introduced in response to the pandemic.^1,2,6,7^ We also included research on initiatives that were active prior to the pandemic since they can also contribute to the knowledge of what works for whom, under which circumstances to reduce FS and improve FWB.

 Research demonstrates FS (or financial dis/stress) – the feeling of not being able to meet financial obligations^8^ – has negative health impacts independent of income^9^ and debt measures.^10^ FWB (or financial health) – that is current and future financial circumstances – also independently impact health.^11-15^ FS and FWB are socially patterned. FS is experienced disproportionately by members of systematically excluded groups, such as African Americans.^16-18^ This has also borne out in relation to the COVID-19 pandemic, which exacerbated FS among many people across the globe.^19-21^

 Prior to the pandemic, FWB and FS initiatives in Canada, the United States, and Australia commonly targeted individual-level behaviour change (eg, building financial literacy or skills).^22,23^ As such, they did not address underlying causes of FS and/or FWB, like the unequal and limited access to secure and sufficient income.^22,23^ Evidence on how to intervene to best address these inequities, however, is not well established. A better understanding of equitable actions to promote the FWB of people and/or households, particularly members of systemically excluded groups, is required to guide governmental and organizational policies and practices in the face of the ongoing global pandemic and ‘recovery’ efforts.

 To contribute to this emerging area of research, we conducted an in-depth critical realist analysis of FWB and FS initiatives in high-income contexts. We identified the underlying architecture that comprises regularities or trends (known as generativemechanisms)^24^ across FS and FWB initiatives in high-income contexts, and how these impact outcomes (ie, what works or does not work), and equity implications (ie, for whom, under what circumstances, why). This study is the first to report on the generative mechanisms of initiatives to address FWB and FS from a public health perspective. It adds a critical, equity-focused viewpoint to a topic area that has until recently been mostly dominated by economists and business scholars.^12,23^

## Methods

###  Theoretical Approach

 Critical realism is a set of philosophical tenants that can be applied to various research methods to identify the underlying drivers (ie, generative mechanisms) of outcomes in real-world settings. We applied critical realist theory to a rapid review of literature to identify and unpack general trends and contextual factors across FS and FWB initiatives.^25^ We used the context-mechanism-outcome configuration to identify the underlying generative mechanisms of FS and FWB in relation to community- and government-led initiatives. In this case, context describes factors within an initiative or the broader context that constrain or enable an outcome (intended or unintended). Mechanisms are the underlying drivers to reasoning or responses that produce a given outcome such as resources or capabilities. Outcomes describe the intended and unintended impact. A generative mechanism highlights forces that underly particular outcomes, including social complexities such as the power and resources that lie with the institutional architecture of society.^24,26^

###  Methodological Approach

 We used rapid review methods^27^ to identify relevant literature for our analysis. A rapid review is a practice-oriented approach to evidence synthesis that is useful to inform decision making. It differs from a systematic review in that the process has been adapted for a shorter time frame^27^ and limited resources while maintaining rigour. This project was part of a larger research-practice collaborative with the aim or creating a practice-ready public health framework and guidebook of strategies and indicators for action on FWB and FS in response to COVID-19. We have reported elsewhere on the detailed methods of the overall project^28^ and the RR descriptive results.^29^

 To identify academic literature, a research librarian conducted a two-concept search (FS and/or FWB AND intervention) using three databases (MEDLINE, PsycINFO, Web of Science Social Science Citation Index). We limited the search to English-language full-text only. For non-peer reviewed literature, we searched two databases (ProQuest, Informit) and filtered search research by source type (reports, other articles). We also conducted searches using Google Advanced with a browser set to ‘private’ mode. The search for non-peer reviewed sources was iterative and we continued until new searches were not retrieving many unique relevant sources.

 We applied the same inclusion and exclusion criteria to the academic and practice-based sources. Inclusion criteria were: (*i*) evaluated an initiative primarily targeting FS or FWB, (*ii*) high-income context,^30^ (*iii*) published in the previous five years (2015–2020), and (*iv*) English-language. Sources were excluded if they were reviews, study protocols, commentaries, editorials, books, or theses. For all sources, we conducted primary screening on the title and abstract and secondary screening on the full text. For academic sources, we conducted tertiary screening where we applied a relevancy criterion and assessed the potential of the source to contribute meaningfully to answering the research purpose, which is in keeping with a realist approach to a rapid review.^31^

 Although we included literature that reported on initiatives introduced in response to the pandemic, we did not limit the literature to these initiatives as is apparent from the 5-year time frame. The purpose of our study was to understand what worked for whom under which circumstances to improve FWB or reduce FS. The initiatives we reviewed did not need to occur during the pandemic to inform future policy and practice, including an equitable recovery.

 We identified 75 relevant practice (n = 39)^2,6,32-65^ and academic sources (n = 36)^22,66-103^ for inclusion. On these sources we conducted an abductive analysis that involved a combination of data extraction techniques (eg, identifying events or occurrences) and critical theorising about that data to describe and interpret them as expressions of more general phenomena.^104^ We developed the coding tools in Epi-Reviewer (academic sources) and then adapted these for use in NVivo (practice literature). We also used Microsoft Word to note preliminary themes and memos.

 The first authors led the analysis of the data they had extracted from either the practice-basedor academic literature.^28^ They created preliminary summaries of their findings outlining context-mechanism-outcome relationships to facilitate collaborative analysis sessions among research team members. These bi-weekly collaborative analysist sessions took place over the course of two-three months. They involved critical discussions and mapping activities using Miro (2021 ed.), a collaborative analysis software. In terms of validity, the generative mechanisms that we report represent conceptual agreement that we achieved through the collaborative analysis process.

## Results

 Our critical realist analysis revealed two distinct ideologies that served as generative mechanisms in FS and FWB initiatives and their outcomes: (1) *neoliberal ideology*, and (2) *social equity ideology*. Additionally, we noted an intersectional generative mechanism, *social location,* which cut across the two ideologies. Therefore, we organize our findings according to the two ideological mechanisms and embed discussions of social location within these.

###  Neoliberal Ideology

 Neoliberalism is a macro-economic and social ideology that has been described and understood in a variety of different ways.^105^ For this study we broadly understood neoliberalism as “a structural force that affects people’s life-chances” and “a system of governance that shapes subjectivities” (p. 89).^106^ More specifically, we conceptualized neoliberal ideology as: a global administrative bureaucracy that promotes and instils free market trade, while simultaneously restricting or eliminating laws that are equity-focused (eg, free healthcare); and, a philosophy of radical individualism in the pursuit of economic capital, that assumes economic behaviour can be understood in terms of the human attributes of rationality, individuality, and self-interest.^107^

 We found that policies, practices, and initiatives based on neoliberal ideology constrained the possibility for systematically excluded groups to experience improved FWB and reduced FS. We noted that the following neoliberally oriented conditions impacted FWB and FS:

1. Restrictive social welfare policies;
2. Limited employment protection policies;
3. Unregulated private housing and insufficient affordable housing policies;
4. Privatized health, elder, and childcare;
5. Exclusionary financial services;
6. Underfunding of community-level initiatives; and,
7. Reliance on individual-level behaviour change.

We detail our findings about what worked and/or did not work, for whom, and why in relation to each of these seven conditions below.

####  1. Restrictive Social Welfare Policies

 We found that initiatives that restricted access to social welfare exacerbated FS and negatively impacted equity. These included: increased pension age^32^; work for welfare programs^34,73,75-77^; tax-exemption-based benefits that prioritize two-parent families^68^; and, breadwinner-based family and labour policies that incentivize ‘traditional’ gender roles and women’s dependence (eg, household taxation, preferential training and employment for men).^82^ Mandatory attendance at job seeking meetings and complex and repetitive administrative requirements with the punitive consequence also failed to improve FWB or reduce FS.^2,37,43,98^ Eligibility requirements for social welfare policies that did not consider people’s complex social locations constrained people’s possibilities to achieve FWB or reduce FS. Among the social locations were noted were Indigenous peoples^34^ – particularly Indigenous peoples living in rural and remote locations,^98^ asylum seekers,^6,52^ people who experience homelessness,^53^ undocumented immigrants,^103^ caregivers,^45,72^ and mothers,^73,79,82^ including single mothers.^43,68^

 Studies in Canada and Australia found that reducing a universal baby bonus and/or replacing it with parental leave policies disproportionately impacted younger and single mothers with low education, income, and employment security compared to women with secure employment and higher income and education.^68,76,77^ The neoliberal restructuring of disability and income support in Australia had a negative impact on the health, social, and FWB of Aboriginal peoples who experience disability living in rural and remote locations (ie, West Kimberley, Australia).^34,98^ We noted a relationship between the conditionality of welfare payments and people accepting precarious employment options (eg, work for welfare programs), which perpetuated reliance on welfare payments and therefore did not achieve the program goals (eg, self-sufficiency) for the target group.^43^ Economic recessions were often driving forces behind austerity-based policies that reduced government spending on social welfare, limited benefits, and created barriers to access,^37^ which disproportionately impacted older women.^72^

####  2. Limited Employment Protection Policies

 Neoliberal labour policies based on individual choice and responsibility promoted precarious employment agreements (eg, casual, part-time, contract-based), did not address structural constraints to employment, provided insufficient leave support, and reduced employment security, which had a detrimental impact on people’s FWB, particularly in the longer-term.^79,83,95,96,102^ This was particularly true for racialized and low-income women,^79,95^ including single mothers,^43^ and people who experienced disability.^83^ Balancing ﬂexible job arrangements and secure transitions between jobs (‘flexicurity’ labour policies), intended to increase employment opportunities by enticing employers with low-risk contracts, did not benefit the employment security or FWB of people with a low education and/or who experience disability.^83^ Similarly, youth entrepreneurship policies enacted in the United kingdom failed to account for the challenges associated with self-employment that were specific to gender, socioeconomic position, and life stage among systematically excluded youth and therefore did not improve their FWB.^102^

 Lack of paid family leave in the US, insufficient duration and timely compensation, and a complicated application processes and information gaps constrained FWB, particularly among low-income mothers^79^ who were racialized and had a low-level of education.^95^ Workers’ compensation schemes in Australia that prioritized return to work and provided insufficient and inadequate compensation (eg, ‘step-down’ programs where compensation decreased over time) increased the FS of people who made claims.^96^ In this case, FS was particularly pronounced among younger workers, family primary earners, and non-standard (eg, casual) employees who did not have access to other forms of income (eg, savings, early retirement).^96^

####  3. Unregulated Private Housing and Insufficient Affordable Housing Policies

 Unregulated private housing markets and weak affordable housing policies are hallmarks of neoliberal policy and commercial determinants of health.^108^ We found that they restricted home ownership, led to high housing costs, and constrained opportunities for FWB among systematically excluded groups.^38,72,78,80,85,93,99^ Rental subsidies in US cities led people to low-quality, inappropriate housing in locations where they felt unsafe and/or lacked amenities to comply with the subsidy terms.^85^ Programs in the US targeting housing affordability and access that had rigid participation requirements – drawing on the neoliberal logic of personal responsibility – often excluded people with multiple social, personal, and economic barriers and/or had high program drop-out.^93,99^ Although improving the FWB and housing security of the small minority of participants who were successful, the underlying philosophy of such programs was apparent in the individual “motivation to change” requirements and the original initiative name: Project Self Sufficiency and Operation Bootstrap.^93^

####  4.Privatized Health, Elder, and Childcare

 We found that the privatized and commercialized (ie, “consumer-” and market-based) care – linked to neoliberal ideology – such as aged care,^32,35,72^ healthcare,^70^ and childcare^76,85,88,95^ constrained people’s opportunities for FWB. In the United Kingdom and Australia, the cost of residential aged care contributed to FS among older people and their families, impacting the effectiveness of these initiatives to enhance FWB.^32,35^ A lack of public or affordable childcare, among other barriers, excluded people, primarily women, from entering or returning to the labour market, limited their access to stable and sufficient employment, and/or rendered them ineligible for related benefits, such as paid parental leave.^73,76,85,95^ The lack of access to, or high cost of, childcare was also cited as contributing to elevated FS. Among families experiencing homelessness a lack of affordable or accessible childcare impeded people’s ability to pursue higher education to improve employment prospects and sustain housing stability.^85^

####  5. Exclusionary Financial Services

 Profit focused banking and financial services, reflecting neoliberal values of free market trade, capital gain, and a lack of state regulatory and protective processes (eg, payday lenders and predatory financers), impacted the effectiveness of initiatives targeting FWB and contributed to FS among systematically excluded groups.^22,33,35,37,44,56,63,77^ In the United States, it was common for participants of financial coaching initiatives to be unbanked.^55,56^ However, initiatives to convert such participants to banking services were limited because deposit and account fees posed barriers to access.^63^ One study in the US targeted financial inclusion (ie, useful and affordable financial services, delivered responsibly and sustainably, to meet people’s needs) through financial coaching. The authors described the initiative as creating new financial consumers (eg, credit-users), which mostly benefited the financial industry rather than the participants.^22^ Moreover, the authors explained that the initiative re-framed the collective and/or structural issue of financial insufficiency (FS, poverty, high cost of living) as a personal issue to be solved by market-based solutions and personal responsibility.^22^

####  6. Underfunding of Community-Level Initiatives

 Restricted funding for FWB or FS initiatives at the community-level impeded their ability to adequately support people/households. A lack of funding adequate also impacted the sustainability of community-level initiatives that had proven effective in reducing FS.^2,37,45,52,67,85^ Funding restrictions to two Australian initiatives providing settlement grants for newly arrived migrants,^52^ and bursary payments to young caregivers,^45^ constrained the ability of these programs to provide tailored and sustainable services. We noted reports of overburdened staff, reflective of insufficient program funding, often arose as a barrier to successful implementation of community-level FWB initiatives.^67,85^ Funding restrictions were often justified in the context of national budget deficits or short-term cost-ineffectiveness; a tenant of neoliberal ideology that prioritizes austerity, balanced budgets, and the interests of a wealthy few over social spending. However, evidence from our reviewed sources did not support this justification. Two examples from Australia included the doubling of the standard social welfare payment due to the COVID-19 economic downturn,^2^ and a comprehensive long-term support initiative for at-risk youth,^37^ both of which reduced FS without an increase in the national budget deficit.

####  7. Reliance on Individual-Level Behaviour Change 

 Behaviour change initiatives reflect the radical individualism of neoliberal ideology, which emphasizes individual responsibility over that of the society. We found such initiatives were common within the literature we reviewed. Yet, we saw no evidence that they improved long-term FWB among systematically excluded groups.^22,35,41,44,56,66,70,73,78,101^ The initiatives did not improve FWB,^22,70,73,78,101^ or resulted in insignificant changes to FWB or FS.^35,40,56^

 Sociocultural tailoring was often absent from the behavioural initiatives that we reviewed. For example, financial literacy programs targeting Indigenous peoples in Australia did not account for the sociocultural obligations people had to lend money to immediate and/or extended family. Such oversight compromised the effectiveness of financial literacy training^40^ and posed barriers to addressing financial insecurity.^44^

###  Social Equity Ideology

 To define social equity ideology, we draw on the principles outlined by Guy and McCandless,^109^ which include: procedural fairness, meaning due process, equal protection and equal rights; equity in the availability and provision of service and benefits; an equal level of outcomes for all population groups; and inclusion of all population groups, particularly groups who experience disadvantage, in decision making regarding policy choices and service delivery.^109^ We found that equity-targeted initiatives mostly reduced FS, and improved equity outcomes, particularly among systematically excluded groups. Specifically, we noted that the following equity-oriented conditions impacted FWB and FS:

1. Strong social welfare policies;
2. protective labour policies;
3. social health, elder, and childcare, housing, and education policies;
4. holistic approaches; and,
5. promotion of community strength and empowerment.

We detail our findings about what worked and/or did not work, for whom, and why in relation to each of these five conditions below.

####  1. Strong Social Welfare Policies

 Social welfare benefits that covered or extended beyond basic needs and had minimal contingencies (eg, work-for-welfare) and barriers to access (eg, extensive assessment) had a positive impact on FWB, particularly among systematically excluded groups.^2,6,33-35,37,43,45,68,76,82,90,92,103^ These included universal-type social equity policies (ie, benefits for all) and targeted-type social equity policies (ie, targeted systematically excluded groups). European countries (ie, EU-27, including the United Kingdom) with social protection-type policies reported higher subjective FWB among residents compared to countries that did not.^90^ Germany’s corporatist protective pension system (eg, publicly funded) promoted greater gender equity later in life for women who followed conventional caregiving trajectories (ie, limited paid work) in comparison to a liberal pension system (ie, contingent on paid work).^82^ The Australian Baby Bonus, a universal benefit, improved the FWB of young mothers.^76^ Targeted-type social support policies improved child poverty rates, reduced income,^68^ and racial^92^ inequalities, and demonstrated a positive cost versus benefit.^37^ In response to COVID-19, Australia doubled the standard social welfare payment. This reduced the number of people living in poverty and the poverty gap,^2^ lowered service usage and housing stress, and improved mental health among recipients.^2,6^

####  2. Protective Labour Policies

 We found that strong labour policies had a beneficial impact on FWB and mostly positive equity impacts.^76,77,79,81,83,88,91,94,95^ Increased minimum wage was one example of such a policy assessed in the United Kingdom. In addition to improving FWB, it benefitted low-wage workers’ overall health, while having no impact on their employment possibilities and hours of work.^81,91^ In comparison to “Flexicurity” (ie, contract-based, casual, part-time) labour policies enacted in Denmark and the United Kingdom, strong employment protection in Sweden and the Netherlands promoted longer-term employment and economic security among people with a low-level of education and/or who experienced disability.^83^

 Parental leave was a supportive labour policy that had the potential to benefit women’s FWB and improve equity, particularly related to gender.^79,88,94,95^ However, few studies we reviewed included parental leave of sufficient duration and compensation. In addition, they had the potential for a negative equity impact because the policies disproportionately benefited higher-income women with secure employment in comparison to lower-income women with more precarious employment circumstances who were often ineligible for the program.^76,77,79,95^

####  3. Social Health, Eldercare, and Childcare, Housing, and Education Policies

 No or low-cost health, eldercare, and childcare and the provision of affordable housing and education reduced FS. This was particularly true among systematically excluded groups,^37,38,45,48,52,53,58,74,80,84,85,88,92,93,97,99^ which aligns with an equitable policy approach. For example, in a study of the 27 EU countries (including the United Kingdom), parents living in countries with generous family benefits, accessible childcare, and flexible work arrangements reported greater life satisfaction and lower financial stress in comparison to parents residing in counties with low levels of support.^88^ An early life education initiative in the United States that targeted neighbourhood disadvantage in addition to education improved the long-term FWB of participants.^92^ An interest-free rental-assistance loan program implemented in a high-deprivation neighbourhood of London, UK, reduced FS and promoted housing security.^80^

####  4. Holistic approaches

 Holistic FWB and FS initiatives provided wrap-around support and/or bundled multiple services into one program (eg, employment and financial counselling).^48,71^ This resulted in simplified access for participants who experienced systemic exclusion, such as single mothers^74^ and at-risk youth.^37,38,58^ For example, in Australia, three initiatives targeting youth exiting out of home care (ie, the child welfare or intervention system), provided financial and housing support combined with close case management were effective in enhancing FWB, and reducing overall stress.^37,38,58^ Overall, we noted that initiatives that were holistic and flexible were effective in enhancing accessibility of FWB-related services and benefits, promoting longer-term FWB, and providing a pathway out of entrenched disadvantage.^33,37,38,48,54,58,71,74,92^

####  5. Promotion of Community-Strength and Empowerment

 FWB initiatives that built or strengthened community connections,^71,78,87,89,100^ capacity,^71,100^ and empowerment, had a positive impact on participants’ social, mental, and physical health,^71,87,89,100^ FWB,^71,87,89^ and economic security.^100^ For example, a Canadian peer-to-peer program that addressed FS-related stigma and built financial literacy skills, enhanced participant optimism, and subsequently reduced FS.^87^ An initiative in the rural US designed and implemented by the White Mountain Apache Tribe used a positive youth development approach to entrepreneurship and was particularly successful at benefiting the community-at-large. It strengthened youth relationships, addressed historical trauma, boosted resiliency, and fostered community pride and sense of belonging.^100^

## Discussion

 We found that neoliberal and social equity ideologies underpinning FWB and FS initiatives impacted their effectiveness. These ideologies also impacted the initiatives’ ability to contribute to and/or achieve equitable outcomes, particularly among systemically excluded groups. For example, more than half of the initiatives reviewed in our study (n = 41) were embedded in individual behaviour change (eg, financial literacy – an approach aligned with neoliberal ideology). While there were few examples that these approaches enhanced short-term FWB (eg, bankruptcy claims among middle class Australians),^66^ effectiveness among systematically excluded groups was limited, particularly in the absence of services for basic needs (eg, housing, education, unemployment). This included for Indigenous or racialized peoples,^34,98^ migrants,^6,52,103^ and single mothers.^43,68^

 The neoliberal globalization of policies has had a negative impact on health and health inequalities across the world,^110,111^ particularly among systemically excluded populations such as Indigenous peoples.^112^ At a societal level (eg, state), austerity policies stem from the economic logic that health and social spending will ‘harm’ the ‘public budget’ or economic growth. They are entrenched in a rhetoric of economic rationality – that is that they make ‘good financial sense.’^113^ However, this rationality or logic is not well-supported by the evidence, including research emerging from the pandemic.^111^ From a social perspective, reduced welfare spending and restrictive policies are associated with increased health inequalities, including worsened mental^114^ and physical health outcomes.^110^ Research from early in the pandemic found that comparing European studies observing unemployment and suicide rates in the general population, contexts with strong social protection such as Sweden show a negative correlation, while countries with or poor social protection such as Italy or Spain show a positive one.^115^ Others have echoed these findings when comparing early pandemic outcomes among countries from across the globe with varying levels of neoliberal policies (eg, public healthcare, funding for social programs).^116^ Furthermore, in a longitudinal comparative study of Canada and the United States, both of which underwent significant neoliberal political reforms from 1980–2008, Canada demonstrated greater equity in the provision of social goods (eg, education) and social cohesion across social locations, which led to greater resilience with regards to health inequalities.^117^

 From a fiscal perspective, evidence has shown that government spending in health and social protection not only improves health equity and contributes to social stability,^111^ but also boosts economic growth.^118^ In their analysis of economic recession (ie, Global Financial Crisis) recovery among high-income economies, Labonté et al^118^ found that increased social spending during economic recession was associated with faster economic and social recovery. Researchers in the United States demonstrated that housing policies (eg, moratorium on rental evictions) introduced to address FS had a positive equity impact on pandemic-related death and illness.^7^ Consideration of the rhetoric versus the reality of policy impacts – eg, ‘good financial sense’ versus actual cost to the social, healthcare, and criminal justice systems – is particularly timely as countries across the globe have implemented COVID-19 recovery plans with a specific focus on economic recovery. The United Nations has put equity at the centre of their Research Roadmap for the COVID-19 Recovery. Many jurisdictions, like Canada,^119^ have committed to an equitable recovery.^120^

 Echoing other research, our findings highlighted the crisis of policies driven by neoliberal ideology.^19,111,121^ Two Australian studies included in our review explored the impact of the government’s decision to double the unemployment benefit (namely ‘Jobseeker’) at the onset of the COVID-19 pandemic (March 2020), reporting a lowering of the national poverty gap by 39%, and a decrease in the total number of people in poverty by 32%^2^; resulting in a decreased burden on social and community services.^6^ The rate returned to the pre-COVID-19 amount in April 2021, despite evidence drawn from economic modeling that it would not be detrimental to the national budget.^2^ This example demonstrates the way power and austerity are used to reinforce neoliberal ideology, assuming that it is socially acceptable for certain systematically excluded groups (eg, chronically unemployed, people living in poverty) to live below the poverty line, and not others (eg, middle class people who lost work or income due to COVID-19). It highlights the need for governments to adopt an equity-focused approach to policies to promote financial recovery from COVID-19 across their populations, particularly among groups who experience disadvantage.^1^ We echo the call from other social science scholars of public policy that a longer-term lens that considers long-standing inequities is required for policy-making now, during our recovery from COVID-19, and into the future.^1,7,122^

 We noted gaps in the literature, which will need to be addressed to fully understand the equity impact of policies and practices on FWB, FS, and related health outcomes. Among the gaps were a dearth of studies focused on Indigenous peoples, people living in rural and remote locations, and racialized and minoritized groups (eg, newcomers). Few studies included the assessment of the longer-term impacts of initiatives, implementation and contextual details, and specific equity impacts, such as how gender, geographical location, race, and the intersection of these (among others) shaped outcomes. To support an equity-approach to COVID-19 recovery-oriented policies and practices, data collection, analysis, and reporting will require a longer-term approach, focus on the differential outcomes for systematically excluded groups, and include implementation and contextual details.^120^

## Conclusion

COVID-19 has had a disastrous impact on the mental, physical, and social health and well-being of populations around the globe,^123^ including people’s FWB.^124,125^ It has been well documented that the negative impacts of COVID-19 have been concentrated among systematically excluded groups, including women, Black folks, Indigenous peoples, and people with a low socioeconomic position.^19,123,126-128^ Amidst the rubble left by the pandemic there is a unique opportunity for change.^120^ Government-led action can move beyond entrenchment in neoliberal ideology^121^ to consider the system as a whole in health policy-making^129^ and improve equity.^7,122,126^ There is also an opportunity for government action to meaningfully support community-led initiatives. For example, governments can ensure adequate and sustainable funding for community initiatives that address the needs, priorities, and preferences of the local populations. Such action will require targeted efforts to harness political will for community-led innovation, addressing the social determinants of health,^130^ and effecting systemic-level change using complex systems approaches.^129^

 In an immediate response to the economic impacts of the pandemic, many governments in high-income countries increased social welfare payments, improved labour protection and unemployment benefits, and addressed access to housing.^3^ That is, they undertook action that aligned with a social equity-based ideology. This demonstrates that it can be done. Early evidence on the impact of such efforts clearly shows their positive impact on people’s FWB and beyond,^131^ specifically in relation to equity.^2,6^ Yet, as governments in high-income countries enter the recovery phase of the pandemic, we are already noticing a scaling back of these policies^5^ and shift in rhetoric in relation from ‘we are in this together’ to ‘getting people back to work’^132^ (ie, to reflect re-alignment with neoliberal values) – rhetoric that reveals the governments’ anxiety that people receiving income supports will continue to rely on assistance. Such an approach diverts attention away from the precarious life circumstances and structural marginalization and oppression of systemically excluded groups and the governments’ responsibility to address these. This is of great concern. If governments fail to take social equity as central to their recovery policies and actions, there is a real risk that the health and social inequities that have long been an issue and were exacerbated by the pandemic will have a lasting negative impact on generations to come.^126^ There is also unique opportunity.^129^ Now is precisely the time to redouble government action and commitments to a different, more fair, and equitable society. Using a social equity ideology to shape FWB and FS policies and related initiatives represents a significant step in the right direction. Listening to the concerns, priorities, and preferences of communities is essential to an equitable recovery. Government support for a bottom-up approach to recovery where communities share the lead, and their initiatives are well-funded and sustainable is another key step toward a more equitable and just future for all.

## Acknowledgements

 We acknowledge Laura Nieuwendyk, Krystyna Kongats, Lisa Allen Scott, Stephanie Montesanti, Jane Springett, Elaine Hyshka, and Roman Pabayo for their contributions to the research project this review was part of. We wish to acknowledge Meghan Sebastianski (PhD) and Diana Keto-Lambert (MLIS) from the Alberta Strategy for Patient-Oriented Research (AbSPOR) SUPPORT Unit Knowledge Translation Platform for their assistance designing and conducting the searches for the academic literature and providing guidance on the practice-based search.

## Ethical issues

 This study did not require ethical approval because the sources of data were published research reports and academic papers.

## Competing interests

 Authors declare that they have no competing interests.

## Authors’ contributions

 NMG and AY: Conceptualization, methodology, formal analysis, visualization, writing–original draft. KJ and AB: Formal analysis, writing–review & editing. EdL: Conceptualization, methodology, resources, writing–review & editing, supervision, project administration, funding acquisition. CIJN and PH: Conceptualization, methodology, resources, writing–review & editing, supervision, funding acquisition.

## Funding

 This work was funded by the Canadian Institutes for Health Research grant number 172694.
